# The Identification of Broomcorn Millet bZIP Transcription Factors, Which Regulate Growth and Development to Enhance Stress Tolerance and Seed Germination

**DOI:** 10.3390/ijms23126448

**Published:** 2022-06-09

**Authors:** Peipei An, Xiang Li, Tianxiang Liu, Zhijie Shui, Mingxun Chen, Xin Gao, Zhonghua Wang

**Affiliations:** State Key Laboratory of Crop Stress Biology for Arid Areas, College of Agronomy, Northwest A&F University, Yangling, Xianyang 712100, China; anpeipei1010@nwafu.edu.cn (P.A.); lixiang199713@nwafu.edu.cn (X.L.); ltxiang@nwafu.edu.cn (T.L.); zhijie_shui@nwafu.edu.cn (Z.S.); cmx786@nwafu.edu.cn (M.C.)

**Keywords:** bZIP transcription factor, gene family analysis, stress response, root development, NAC transcription factor

## Abstract

Broomcorn millet (*Panicum miliaceum* L.) is a water-efficient and highly salt-tolerant plant. In this study, the salt tolerance of 17 local species of broomcorn millet was evaluated through testing based on the analysis of the whitening time and the germination rate of their seeds. Transcriptome sequencing revealed that *PmbZIP131*, *PmbZIP125*, *PmbZIP33*, *PmABI5*, *PmbZIP118*, and *PmbZIP97* are involved in seed germination under salt stress. Seedling stage expression analysis indicates that *PmABI5* expression was induced by treatments of high salt (200 mM NaCl), drought (20% W/V PEG6000), and low temperature (4 °C) in seedlings of the salt-tolerant variety Y9. The overexpression of *PmABI5* significantly increases the germination rate and root traits of *Arabidopsis thaliana* transgenic lines, with root growth and grain traits significantly enhanced compared to the wild type (Nipponbare). BiFC showed that PmABI5 undergoes homologous dimerization in addition to forming a heterodimer with either PmbZIP33 or PmbZIP131. Further yeast one-hybrid experiments showed that *PmABI5* and *PmbZIP131* regulate the expression of *PmNAC1* by binding to the G-box in the promoter. These results indicate that *PmABI5* can directly regulate seed germination and seedling growth and indirectly improve the salt tolerance of plants by regulating the expression of the *PmNAC1* gene through the formation of heterodimers with *PmbZIP131*.

## 1. Introduction

Cereals are the most important sources of carbohydrate acquisition and are the most widely planted crops in global agriculture. On a worldwide basis, the main crops grown in Asia are maize (*Zea mays* L.), wheat (*Triticum aestivum* L.), broomcorn millet (*Panicum miliaceum* L.), and sorghum (*Sorghum bicolor* L.); rice (*Oryza sativa* L.), barley (*Hordeum vulgare* L.), and foxtail millet (*Setaria italica* L.) are the most popular crops produced in the Americas, while rye (*Secale cereale* L.) and rice are heavily cultivated in Europe [1]. In 2018, United States cereal production was 8.69 tons per hectare, while that of China was only 6.08 tons per hectare [2]. In a situation of global climate extremes, land degradation, and decreasing amounts of cultivated land, adverse factors can cause a range of morphological, physiological, and biochemical damage to plants, resulting in serious losses to agricultural production and economic yields [3]. Therefore, improving the tolerance of crops to unsuitable ecological conditions (i.e., abiotic stress and biotic stress) is a persistent goal of plant breeders and researchers [4].

Abiotic stresses represent abiotic environmental conditions that are harmful to plant survival, growth, and development, even leading to injury, damage, and death. The main adverse environmental conditions that plants often encounter are extreme temperatures, drought, salinity, and heavy metal toxicity [5]. Drought stress can occur at any stage of crop growth and development and last for varying periods of time with varying intensities [6]. It has been revealed that plants are able to adjust the development of their roots to grow thicker and deeper, which is an important mechanism for avoiding drought stress [7]. Salt stress is also one of the major environmental stresses that limit plant growth and productivity, and plants can adapt to salt stress through flexible regulation of hormone levels and signaling [8]. Roots are the key organ for absorbing water and nutrients in plants [9]. The rate at which lost plant water is replaced is an important indicator of plant salt tolerance, and it is affected not only by root length and root diameter but also by the root cortex width, number of root hairs, and number of xylem vessels [10,11,12]. Under salt stress, plants with a higher proportion of roots are more able to facilitate the retention of toxic ions within this organ and control their transport to the aboveground part [13]. This may be a typical resistance/survival mechanism in plants under saline conditions [14,15]. In addition, extreme temperatures can be recognized by plant temperature sensors, and this information then transduced to a regulatory network that initiates multiple responses [16]. The toxicity of heavy metals affects plant growth and development, leading to reduced photosynthesis, changes in enzyme activity, and resulting in oxidative damage [17,18,19] that, when accumulated in plants, can threaten the health of animals and human beings that eat these cereals [20]. Therefore, future research needs to be informed using an integrated omics database to study abiotic stress mechanisms in combination with transgenic means to improve crop stress resistance.

Members of the basic leucine zipper (bZIP) transcription factor (TF) family are known to function in plant development, pathogen defense, and light and stress signaling response. bZIP TFs usually function via hetero- or homodimerization, which provides huge combinatorial potential for gene expression control and signal integration [21]. bZIP TFs prefer to bind to the A-box (TACGTA), C-box (GACGTC), and G-box (CACGTG) elements in the promoter region, but there are also instances of non-echo binding sites [22,23,24]. *ABA INSENSITIVE 5* (*ABI5*), one of the group A members of the bZIP TF family, plays a vital role in regulating seed germination, root meristem growth, and seedling growth, modulating abscisic acid (ABA) and auxin responses [25,26,27,28]. *ABRE-BINDING FACTORS 1* (*AREB/ABF2*) in the bZIP TF subfamily A can be upregulated by ABA and water stress and interact with *SRK2D/SnRK2.2*, an SnRK2 protein kinase, to regulate ABRE-dependent gene expression for ABA signaling [29]. However, the function of *ABF2* and *ABI5* in broomcorn millet remains elusive.

Next-generation sequencing (NGS) technology is based on sequencing by synthesis, which is able to reflect the RNA transcript level of genes in characterizing expression. NGS has increasingly been used as a preliminary tool for exploring gene function and even biological questions in recent years [30]. The main aim of this study, based on a combination of Illumina NGS, a seed germination experiment, and NGS analysis, is to investigate the salt tolerance mechanisms operating during broomcorn millet germination. The research explores the genes responsible for salt tolerance and the correlation with transcription factors from Yumi 1 (Y1) and Yumi 9 (Y9) broomcorn millet varieties. Moreover, the results of the broomcorn millet seed germination are compared and analyzed with and without salt treatment to explore the molecular aspects of salt tolerance in this species. This study plays an important role in improving our understanding of stress tolerance for application in molecular breeding. A large number of salt-tolerant-related genes and seed germination genes were identified in this work, which provides a theoretical basis for the participation of bZIP TF in stress response in broomcorn millet.

## 2. Results

### 2.1. Seed Germination Rate in Salt Treatment

The seeds of all varieties of broomcorn millet germinated on Day 3 in RO water, but those of Longmi 7, Neimi 5, Yumi 8, Xingmi 1, and Yanmi 7 did not germinate on the third day under NaCl (250 mM) treatment (Table 1). Observations of broomcorn millet seeds under NaCl (250 mM) were discontinued after 7 days, since no seeds had germinated by that time, and the remaining seeds were probably nonviable (i.e., they were no longer firm). The germination rate of Yumi 9, Yanmi 7, and Yanmi 8 were 100%, 96%, and 95% in CK, respectively. However, several varieties of broomcorn millet had poor germination ability in CK, such as Qingmi 1 and Ningmi 10. When the seeds were subjected to 250 mm NaCl treatment, the Xingmi 1 germination rate dropped sharply to only 2%; the germination rate of Qingmi 1 and Qingmi 2 decreased significantly, to 6% and 25%, respectively. The other low-germination-rate variety was Yumi 1, where only 23 seeds germinated. It is worth noting that a higher germination rate was maintained by Yumi 9 seeds, above 70%. Therefore, we preliminarily determined that Yumi9 is a salt-tolerant variety, while Xingmi 1 and Yumi 1 are salt-sensitive varieties.

On the basis of the sequencing scheme set to identify varieties demonstrating tolerance to extremes of the two treatments in three periods, it was found, after preliminary experiments, that there were three nodes in the process of seed germination, with the following periods: 4 °C culture results in total seed inhibition; 25 °C 3 h after germination, expression of early salt-stress-related genes begins; 25 °C when cultivating 6 to 24 h after seed germination, a large number of seed-germination- and salt-stress-related genes are expressed. Considering that the germination rate of Xingmi 1 is too low to allow reaching the sample quantity required for RNA-Seq analyses, Yumi 9 and Yumi 1 were confirmed as the sample varieties.

### 2.2. Transcriptome Sequencing and Assembly

To fully characterize the transcriptome and gene expression levels of *P. miliaceum* under water (CK) and salt stress (NA) in the three periods, 36 cDNA samples of broomcorn millet seeds were prepared and sequenced using the Illumina HiSeq TM platform. The RNA-Seq raw data are summarized in Table 2, whereby the results are the averages of three replicates. After removing low-quality and adapter-related reads, 317.55 Gb clean reads were obtained, with an average of 8.82 Gb (29.5 million) reads for each sample, and the percentage of Q30 base in each sample was not less than 85.06%. The clean data of each sample were compared with the reference genome. The ratio of mapped reads to the reference genome ranged from 72.93% to 84.57%, and unique mapped reads ranged from 71.51% to 82.51%.

### 2.3. Gene Annotation and Functional Classification

All gene sequences were queried to screen databases such as COG, GO, KEGG, KOG, SwissProt, Pfam, eggNOG, and Nr, after which the genes were annotated with the corresponding information identified. On the basis of the sequencing results (Table 3), 18,435 genes (30.56%) were annotated in COG, 44,154 genes (73.19%) in GO, 17,925 genes (29.71%) in KEGG, 28,998 genes (48.06%) in KOG, 47,074 genes (78.03%) in Pfam, 37,227 genes (61.70%) in Swiss-Prot, 56,482 genes (93.62%) in eggNOG, and 59,971 genes (99.40%) in nr. There were about 60,331 genes annotated in total, of which 24,955 genes were longer than 300 bp and shorter than 1000 bp, and 32,893 genes were longer than 1000 bp.

**Figure 1:**
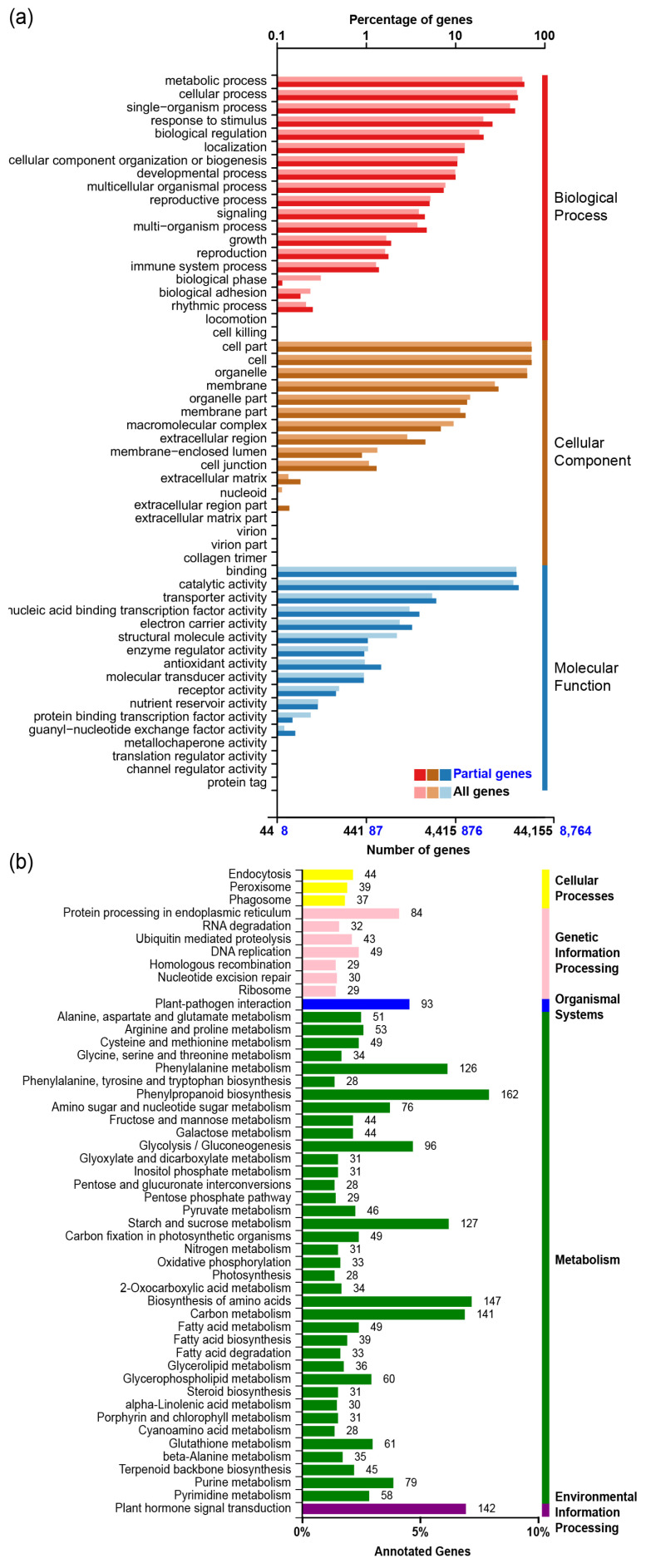

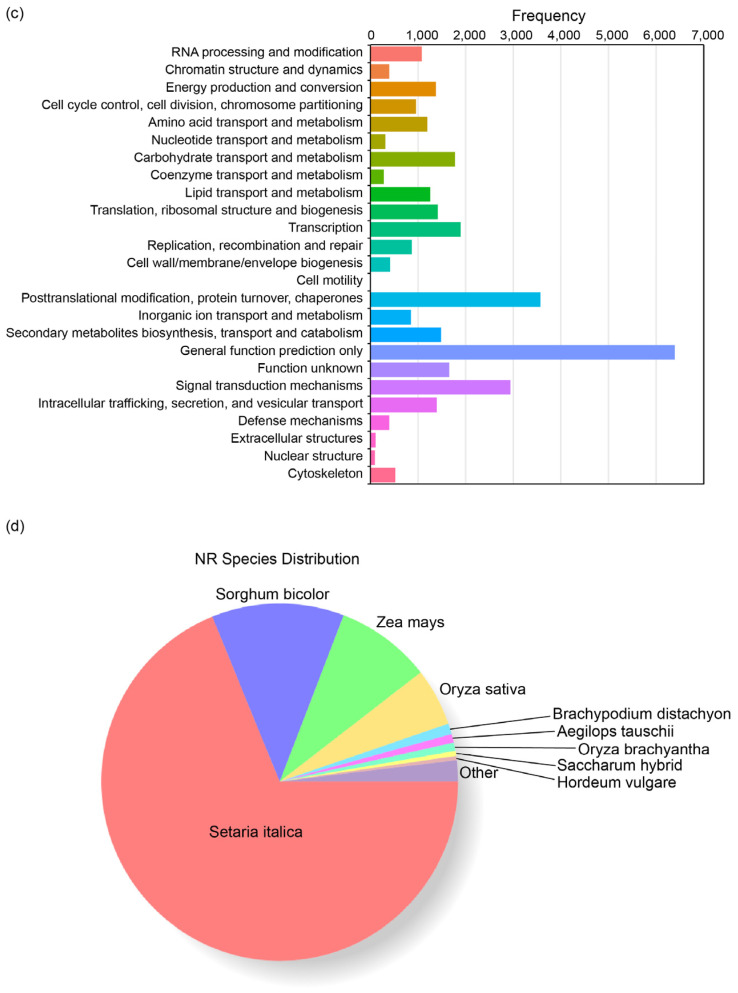
GO, KOG, NR, and KEGG annotation. (**a**) GO annotation to demonstrate the possible functional classifications of broomcorn millet genes. The lighter bars correspond to all genes (black), and the darker bars to differentially expressed genes (DEGs, blue). The genes were categorized into three main types: “biological process”, “cellular component”, and “molecular function”. (**b**) KEGG annotation showed that most of the genes were in the metabolism category. The genes were grouped into “cellular process”, “genetic information processing”, “organismal systems”, “metabolism”, and “environmental information processing”. (**c**) KOG annotation grouped all genes into 25 functional classifications. The number of genes annotated for this function and the percentage of total genes are shown in square brackets. (**d**) nr annotation showed that most of the sequenced broomcorn millet genes are highly similar to corresponding genes in *Setaria italica* and *Sorghum bicolor*.

Gene Ontology (GO) annotation was used to demonstrate the possible functional classifications of broomcorn millet genes. Successfully annotated and classified genes were distributed into 54 functional groups in the categories of cell component (CC), molecular function (MF), and biological process (BP) (Figure 1a). The biological process category covered 22,969 genes and was successfully identified in 20 subgroups, where most annotations were in “metabolic process” (5164, 22.5%), “cellular process” (4369, 19.0%), “single-organism process” (4070, 17.7%), and “response to stimulus” (2268, 9.9%); 24,226 genes were identified in 17 subgroups for the cell component category, where the top four GO subgroups were “cell part” (6249, 25.8%), “cell” (6237, 25.7%), “organelle” (5550, 22.9%), and “membrane” (2646, 10.9%); and 10,322 genes were annotated in 17 subgroups for the molecular function category, where the top three GO terms were “catalytic activity” (4457, 43.2%), “binding” (4225, 40.9%), and “transporter activity” (532, 5.2%). KEGG annotation showed that most of the genes were in the metabolism category (Figure 1b), and many genes were in the environmental information processing category, which is mainly enriched in signal transduction genes (including those participating in plant hormone signal transduction and the phosphatidylinositol signaling system). According to the KOG annotation, 28,998 genes were grouped into 25 functional classifications (Figure 1c). Around one-fifth of genes were classified as “general function prediction only” (6391, 19.59%), and fewer as “function unknown” (1655, 5.07%). Aside from this, the top three groups were “posttranslational modification, protein turnover, chaperones” (3572, 10.95%), “signal transduction mechanisms” (2939, 9.01%), and “transcription” (1894, 5.81%). The nr database had identified the most annotated genes of the databases used in searching (Figure 1d). According to the results, *P. miliaceum* showed the most matches to *Setaria italica* (41,276, 68.83%), followed by *Sorghum bicolor* (7199, 12.01%), and *Zea mays* (5196, 8.67%).

### 2.4. Statistics of Differential Expression Genes (DEGs) in Salt Stress

The twelve groups of differential expression genes (DEGs) are summarized in Table 4, where it can be seen that a large number of DEGs were identified in the groups of Y9_CK3 vs. Y9_NA3 and Y1_CK3 vs. Y1_NA3, including 2860 upregulated and 1456 downregulated genes and 1447 upregulated and 1604 downregulated genes, respectively. In Y9_CK2 vs. Y9_NA2, 1898 DEGs (613 up- and 1285 downregulated) were identified, compared with Y1_CK2 vs. Y1_NA2 (97 up- and 328 downregulated), showing that the response to salt stress in Yumi 9 was earlier. Further research revealed that the number of genes found to be differentially expressed between Yumi 1 and Yumi 9 was relatively stable in the sampling stages 2 and 3, and the numbers of DEGS were 2442 (Y1_CK2 vs. Y9_CK2) and 1968 (Y1_CK3 vs. Y9_CK3), respectively. However, the number of DEGs decreased to 1406 (Y1_NA2 vs. Y9_NA2) and 1338 (Y1_NA3 vs. Y9_NA3) after NaCl treatment.

**Figure 2:**
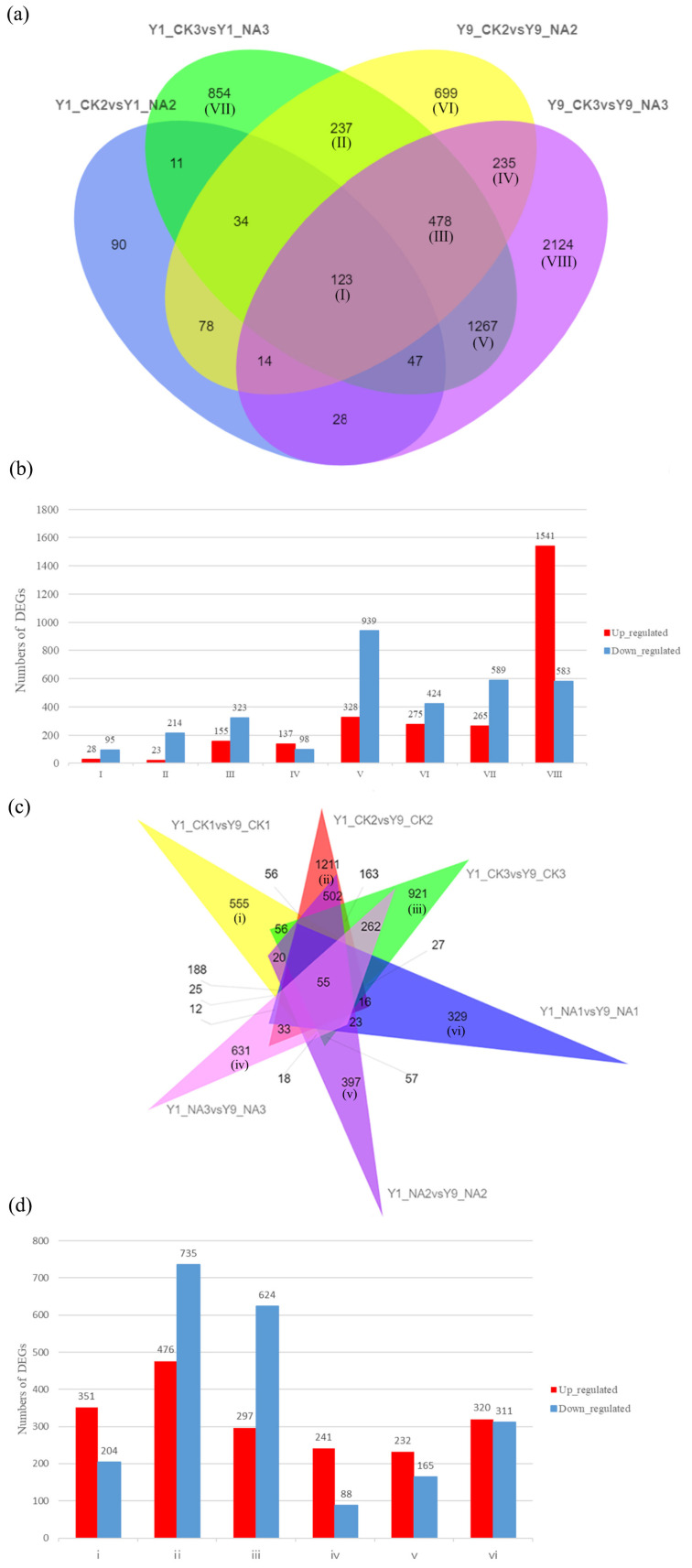
**Venn analysis and bar charts of DEGs during salt stresses.** (**a**) Venn diagram of DEGs due to 250 mM salt treatment. (**b**) Number of upregulated and downregulated genes corresponding to each part of (**a**). (**c**) Venn of DEGs caused by salt resistance. (**d**) Number of upregulated and downregulated genes corresponding to each part of (**c**). I–V indicate overlapped DEGs between Y1_CK2 vs. Y1_NA2, Y1_CK3 vs. Y1_NA3, Y9_CK2 vs. Y9_NA2 and Y9_CK3 vs. Y9_NA3; Y1_CK2 vs. Y1_NA2 and Y9_CK2 vs. Y9_NA2; Y1_CK3 vs. Y1_NA3, Y9_CK2 vs. Y9_NA2 and Y9_CK3 vs. Y9_NA3; Y9_CK2 vs. Y9_NA2 and Y9_CK3 vs. Y9_NA3; Y1_CK3 vs. Y1_NA3 and Y9_CK3 vs. Y9_NA3, respectively, in (**a**,**b**). VI–VIII indicate unique DEGs in group Y9_CK2 vs. Y9_NA2, Y1_CK3 vs. Y1_NA3 and Y9_CK3 vs. Y9_NA3, respectively. i–vi indicate unique DEGs in group Y1_CK1 vs. Y9_CK1, Y1_CK2 vs. Y9_CK2, Y1_CK3 vs. Y9_CK3, Y1_NA3 vs. Y9_NA3, Y1_NA2 vs. Y9_NA2 and Y1_NA1 vs. Y9_NA1, respectively, in (**c**,**d**).

Moreover, the Venn diagram in Figure 2a displays unique and overlapping sets of DEGs under salt treatment. There were 123 DEGs (I, 28 up- and 95 downregulated, Figure 2b) expressed in all comparisons, demonstrating that these genes were shared and function continuously in response to salt stress. A majority were expressed in the third stage of sampling under salt stress, 1267 DEGs were marked as overlapping (V, 328 up- and 939 downregulated), indicating that these genes showed a relatively late response to salt. There were 235 DEGs (IV, 137 up- and 98 downregulated) in Yumi 9 that specifically responded to salt stress; however, there were only 11 DEGs in Yumi 1. We also identified 699 (V, 275 up- and 424 downregulated), 854 (VII, 265 up- and 589 downregulated) and 2124 (VIII, 1541 up- and 583 downregulated) unique genes differentially expressed only in Y1_CK3 vs. Y1_NA3, Y9_CK2 vs. Y9_NA2 and Y9_CK3 vs. Y9_NA3, respectively.

The Venn diagram in Figure 2c presents unique sets of DEGs in two extreme-tolerant plant varieties, suggesting that these genes may be responsible for the variety differences in terms of salt tolerance. The two groups with the highest numbers of DEGs were treated with RO water; the 1211 DEGs (476 up- and 735 downregulated, Figure 2d) were uniquely expressed in the second stage of sampling (Y1_CK2 vs. Y9_CK2, ii). In the third stage of sampling, they were treated using RO water (Y1_CK2 vs. Y9_CK2, ii), after which 921 DEGs (297 up- and 624 downregulated) were uniquely expressed. However, under salt stress, the number of differentially expressed genes was the highest in the third stage (Y1_NA3 vs. Y9_NA3, vi), where there were 351 upregulated and 204 downregulated DEGs. The number of differentially expressed genes was the lowest in the first stage (Y1_NA1 vs. Y9_NA1, iv), where there were 241 upregulated and 88 downregulated DEGs.

### 2.5. GO Term and KEGG Enrichment Analysis for DEGs

**Figure 3:**
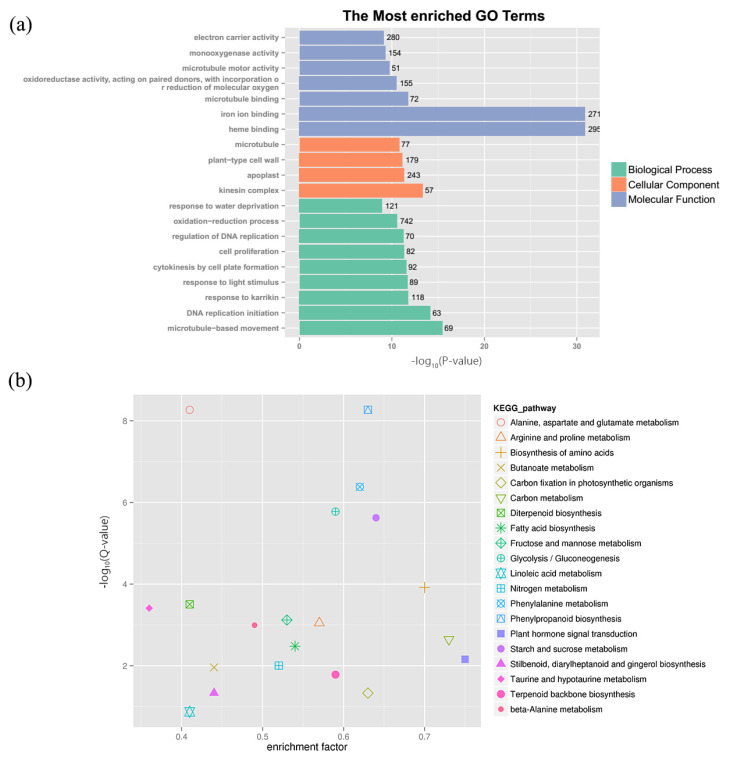
GO enrichment and KEGG enrichment of DEGs (Y9_CK3 vs. Y9_NA3). (**a**) Top 20 GO term DEGs were enriched in three main categories: “biological process”, “cellular component”, and “molecular function”. (**b**) KEGG enrichment of DEGs. The horizontal axis represents enrichment factor, and the vertical axis represents Q-value. Different markers represent different pathways.

Gene enrichment analysis uses predefined sets of genes and sequences to identify significant biological changes or patterns of gene co-expression, and it can assess the functional relevance of target sets of genes derived from a set of experiments [31]. The most enriched GO terms were classified into 20 functional groups in the following categories: cell component (CC, 4 groups), molecular function (MF, 7 groups), and biological processes (BP, 9 groups) (Figure 3a). Among them, DEGs were mostly enriched in the oxidation–reduction process (742, belonging to biological process), heme binding (295, belonging to molecular function), and electron carrier activity (280, belonging to molecular function). The KEGG analysis results showed that DEGs were mostly enriched in phenylpropanoid biosynthesis (162, ko00940), plant hormone signal transduction (142, ko04075), carbon metabolism (141, ko01200), starch and sucrose metabolism (127, ko00500), and phenylalanine metabolism (126, ko00360) (Figure 3b).

### 2.6. Expression Analysis of TFs Involved in Seed Germination under Salt Stress

**Figure 4:**
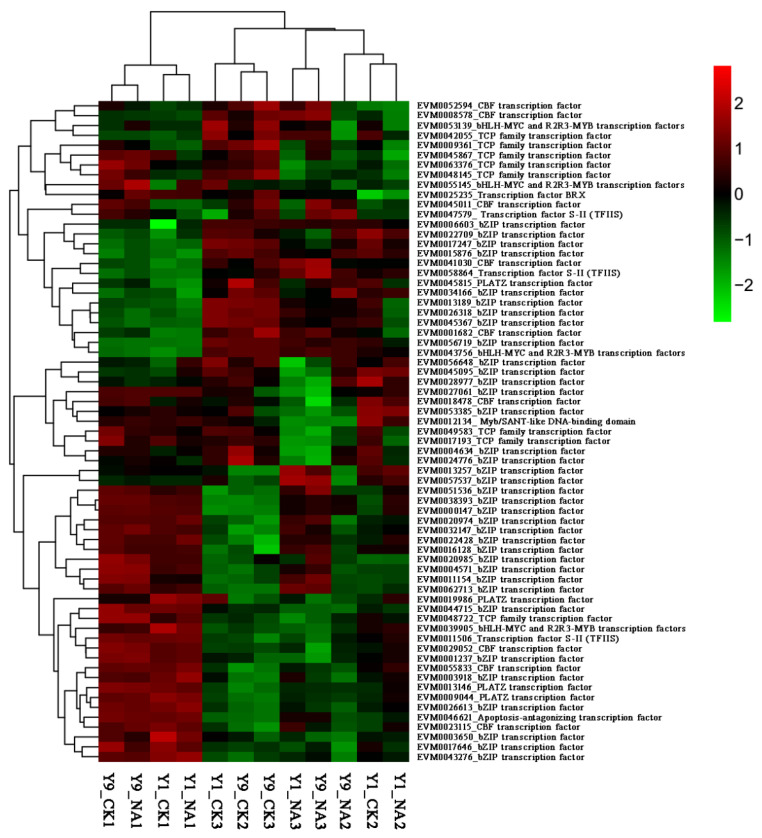
Heatmap of TFs involved in seed germination under salt stress. Heatmap of TFs were clustered by columns. Y9, Y1 represent Yumi 9 and Yumi 1, respectively. CK represents control, NA represents salt stress (250 mM NaCl). 1, 2, 3 represent the three periods. Y9_CK1 refers to Yumi 9 germination under RO water in the first period. The treatments and varieties are similarly indicated in the remaining labels. The information on the righthand side of the heatmap corresponds to the gene ID and annotation.

Seed germination marks the beginning of a new growth cycle of higher plants, and it is affected by complex regulatory mechanisms of internal and environmental signals [32]. In eukaryotes, the synergy between transcription factors and the core transcriptase RNA polymerase initiates gene expression [33]. We comprehensively analyzed the transcription level changes of different expression TFs in seed germination under salt stress (Figure 4). The heatmap revealed that a large number of bZIP transcription factors (36 *PmbZIPs*) exhibit differences in expression during this period, which means that they are involved in the germination of seeds under salt stress. In addition, the transcription factors (TFs) of other families mainly include CBF transcription factor (members of the AP2/ERF family), TCP transcription factor, MYB transcription factor, and PLATZ transcription factor (plant AT-rich protein and zinc-binding protein). It is worth noting that *EVM0016128* (*PmbZIP131*, *PmABF2*), *EVM0022428* (*PmbZIP125*), *EVM0000147* (*PmbZIP33*, *PmABF4*), *EVM0038393* (*PmbZIP30*, *PmABI5*), *EVM0059197* (*PmbZIP118*, differential expression, but very low), *EVM0045095* (*PmbZIP97*), and *EVM0056648* (*PmbZIP89*) were differentially expressed. Of these 7 PmbZIP transcription factors, 5 *PmbZIPs* (*PmbZIP131*, *PmbZIP125*, *PmbZIP33*, *PmABI5*, *PmbZIP118*) were upregulated by salt treatment and 2 *PmbZIPs* (*PmbZIP89*, *PmbZIP97*) were downregulated.

### 2.7. PmbZIPs Response to Various Stress Conditions in the Plant Seedling Stage

**Figure 5:**
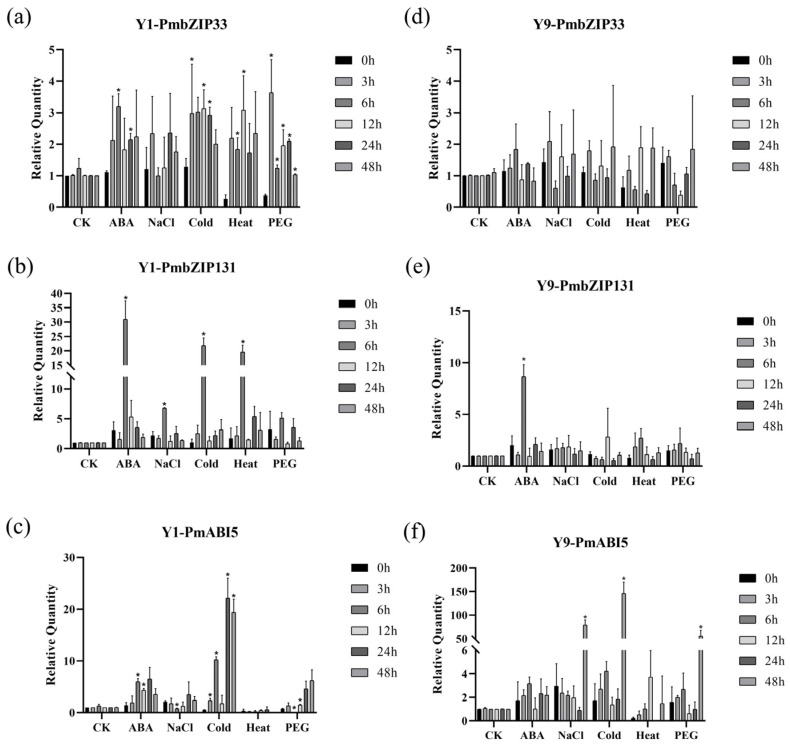
Expression pattern of *PmbZIPs* in the seedling stage. Error bars are SD based on three biological replicates and three technical repeats. *: *p* value < 0.05. (**a**) Expression levels of *PmbZIP33* in Y1. The horizontal axis represents treatment with RO water (CK), 100 μM ABA (ABA), 200 mM NaCl (NaCl), 4 °C cold incubation (Cold), 42 °C heat stress (Heat), and 20% PEG6000 (PEG). (**b**) Expression levels of *PmbZIP131* in Y1. (**c**) Expression levels of *PmbABI5* in Y1. (**d**) Expression levels of PmbZIP33 in Y9. (**e**) Expression levels of *PmbZIP131* in Y9. (**f**) Expression levels of PmbABI5 in Y9.

To explore the response of seven *PmbZIP* transcription factors to different stresses, we analyzed the expression patterns using quantitative real-time reverse transcription (qRT)-PCR. The experimental results showed that *PmABI5* could be significantly induced by salt, drought, and low temperature in Y9 millet after treatment for 48 h (salt-tolerant) (Figure 5f). Beyond this, expression of *PmABI5* was also rapidly induced by ABA in Y1 millet when treated for 6 h (salt-sensitive), indicating that the two varieties possess different patterns of gene expression corresponding to differences in regulation (Figure 5e). Meanwhile, *PmbZIP131* was induced by ABA, low temperature, and high temperature in Y1 (salt-sensitive), while there were no obvious expression changes in Y9 (Figure 5b,e). This result shows that the gene has different expression patterns in the two selected varieties. *PmbZIP33*, *PmbZIP125*, and *PmbZIP118* exhibited no expression change under most treatments (Figure S1). Although *PmbZIP33* showed significant differences after treatment, the expression level was relatively low (Figure 5a). Two U-subfamily genes (*PmbZIP97* and *PmbZIP89*) were strongly induced by ABA, salt, low temperature, high temperature, and drought stress in Y1 millet (Figure S1). These results are consistent with the previous germination stage by RNA-Seq, indicating that *PmbZIP33*, *PmbZIP131*, and *PmABI5* are upregulated under salt stress. However, the fact that *PmbZIP89* and *PmbZIP97* were upregulated in the seedling stage may be due to inhibition of regulation at a specific time.

### 2.8. PmbZIPs Subcellular Localization and BiFC Assay

**Figure 6:**
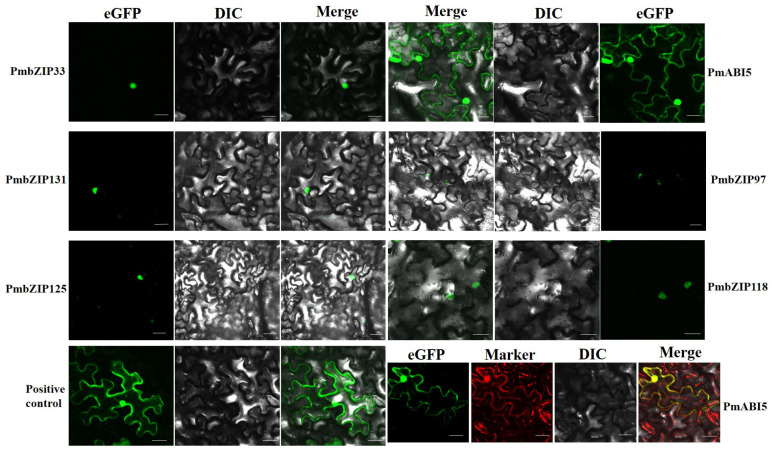
Subcellular localization of PmbZIPs. Marker was an endoplasmic reticulum (ER) marker protein; the positive control was pCAMBIA2300-eGFP. Red color indicates ER marker, green color indicates eGFP signal. Scale bars = 50 μM.

The PmbZIP proteins were fused with enhanced green fluorescent protein (eGFP) at the N-terminus and transiently expressed in *N. benthamiana* leaf via *Agrobacterium*-mediated transformation, and the eGFP signal showed exclusive nuclear localization in all cases except for PmABI5 (Figure 6). Fluorescent signals corresponding to PmABI5:eGFP transiently expressed in *N. benthamiana* were found in both the nucleus and the endoplasmic reticulum.

**Figure 7:**
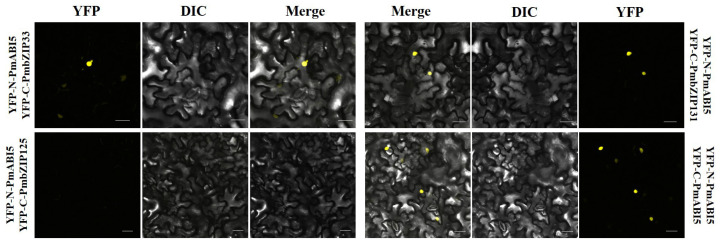
Bimolecular fluorescence complementation of *PmbZIPs*. Yellow color indicates YFP signal. Scale bars = 50 μM.

To further confirm the possibility of a direct interaction, we performed bimolecular fluorescence complementation (BiFC) assays. YFP-N-*PmABI5* and YFP-C-*PmbZIP33* were co-transformed into *Nicotiana benthamiana* leaves, a strong yellow fluorescent signal was observed in the nucleus (Figure 7). YFP-N-PmABI5 and YFP-C-PmbZIP131, as well as YFP-N-PmABI5 and YFP-C-PmABI5, had the same phenotype. These results indicate that PmABI5 directly interacts with PmbZIP33, PmbZIP131, and PmABI5 in vivo, forming homodimers or heterodimers in order to function.

### 2.9. PmbABI5 Positively Regulates Root Growth in Arabidopsis and Rice

**Figure 8:**
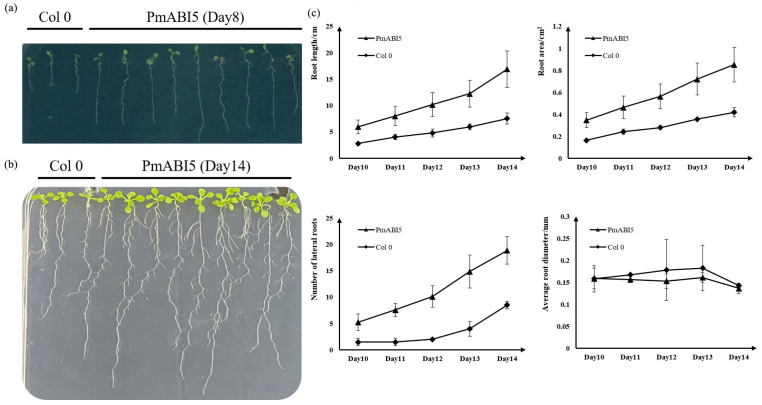
Identification of root traits of *PmABI5 Arabidopsis* overexpressing transgenic lines. (**a**) OE:*PmABI5* Root Phenotype on Day 8 of germination. (**b**) OE:*PmABI5* Root Phenotype on Day 14 of germination. (**c**) Mean values of OE:*PmABI5* root length, number of lateral roots, root surface area, and average root diameter. Error bars indicate standard deviation, sample size of PmABI5 corresponds to the 9 transgenic *Arabidopsis* lines in Figure 8b, sample size of Col 0 corresponds to the 3 *Arabidopsis* lines in Figure 8b.

To investigate the possible role of *PmABI5* in salt stress during germination and root elongation, we analyzed the germination rate and root growth in *Arabidopsis* *PmABI5* overexpression lines (OE: *PmABI5-3-1*; OE: *PmABI5-4-6*; and OE: *PmABI5-7-4*) cultured under 0 and 100 mM NaCl. Under salt-free conditions, the germination rates of *PmABI5* overexpression lines and the wild type were the same (Figure S2), but root length, root area, and lateral root number were significantly higher than those of the wild type (Figure 8). Moreover, the germination rate and root development of *PmABI5* overexpressing plants were also significantly higher than those of wild type on medium containing 100 mM NaCl (Figure S2).

**Figure 9:**
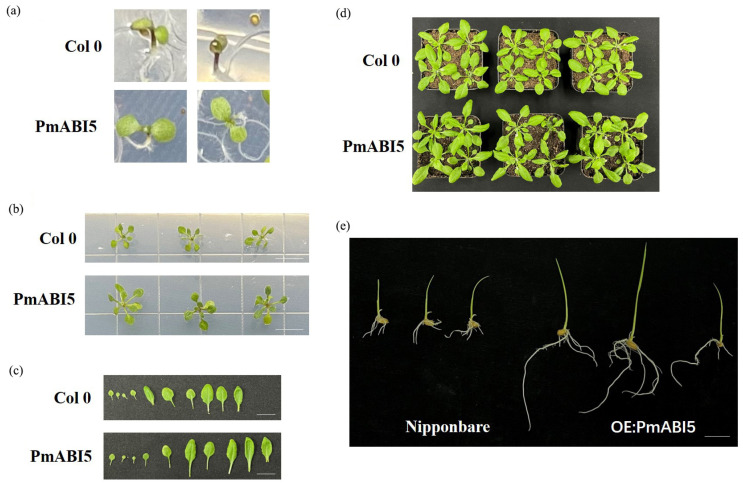
Identification of root traits of transgenic *Arabidopsis* *PmABI5*-overexpressing lines. (**a**) OE:*PmABI5* alleviates plant stress. (**b**) 15-day-old OE:*PmABI5* and Col-0. Scale bars = 1 cm. (**c**) The leaf phenotype of germinated 6-week OE:*PmABI5*. Scale bars = 2 cm. (**d**) The whole plant phenotype of germinated 6-week OE:*PmABI5*. (**e**) Root phenotype of *PmABI5* rice overexpression lines. Scale bars = 1 cm.

**Figure 10:**
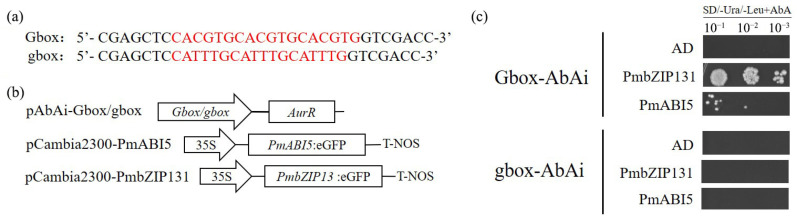
Single hybridization of PmABI5 with G-box in yeast. (**a**) Structures of G-box and g-box in the pAbAi vector. Letters in red represent the three copies of G-box (CACGTG) and the mutanted sequence g-box (CATTTG). (**b**) Effector and reporter constructs used in yeast one-hybrid assay. (**c**) PmABI5 and PmbZIP131 bind directly to G-box.

It is worth noting that leaves of the wild type under salt stress had already started to show signs of stress (anthocyanin accumulation), while the overexpression lines still did not exhibit the stress phenotype at 8 days old (Figure 9a). For further confirmation of our hypothesis, the 15-day-old *PmABI5*-overexpressing lines and wild-type seedlings were transferred to soil (Figure 9b). After a further 4 weeks of growth, *PmABI5* overexpression lines had the same number of rosette leaves as the wild type, but with significantly larger leaves (Figure 9c,d). Figure 10e shows 7-day-old seedlings of rice, and *PmABI5*-overexpressing rice lines have the same phenotype as the corresponding *Arabidopsis* line in terms of root development. The seed size of the transgenic strain after harvest was also larger than that of Nipponbare (Figure S3).

### 2.10. PmbABI5 Can Regulate PmNAC1 Expression

To delineate the exact sequence of the G-box motifs in the *PmNAC1* promoter that are bound by PmABI5, three copies of G-box (CACGTG) and its mutant sequence g-box (CATTTG) were cloned into the pAbAi vector (Figure 10a,b). Then, they were transferred to the yeast Y1H strain after linearization to obtain Gbox-AbAi and gbox-AbAi strains: AD (empty pGADT7 vector), AD-PmbZIP131 (full-length *PmbZIP131* fused to GAL4 activation domain), and AD-PmABI5 via transformation of each as newly constructed strains. Our results show that both PmABI5 and PmbZIP131 were able to bind to the wild-type G-box, but not the mutant (in which CACGTG was mutated to CATTTG) (Figure 10c). Furthermore, the strength of PmbZIP, at 131 interactions, was higher than that of PmABI5.

## 3. Discussion

*PmABI5* is induced by various stresses and is presumably involved in the regulation of multiple stress responses. Several studies have demonstrated that this family of genes can improve plant tolerance to the corresponding stresses by regulating the expression of key resistance genes [34,35,36]. Several studies have highlighted the involvement of ABI5 in seed germination and post-germinative growth, suggesting that it may interact with JA to mediate these processes in *Arabidopsis thaliana* and rice [37,38,39]. Moreover, previous research has revealed that ABI5 regulates the expression of PYL11 and PYL12 by directly binding to their promoters, and PYL11 and PYL12 positively modulate ABA-mediated seed germination and seedlings [40]. ABI5 protein can heteromerically interact with HY5 to activate its own expression, regulating the adaptation of young seedlings to environmental stresses [41]. However, the detailed molecular mechanisms underlying ABI5 regulation of seed germination and post-germinative growth in salt-tolerant millet remained elusive. In this study, we further investigated the regulatory role of *PmABI5* in modulating ABA and salt responses during seed germination and seedling growth. The results show that *PmABI5* can be induced by salt, ABA, heat, and drought, heteromerically interacting with *PmbZIP131* or homologously interacting with itself to promote seed germination under salt stress. In addition, overexpressing *PmABI5* in *Arabidopsis thaliana* and rice can significantly increase seed germination rate and root development under saline stress. These results are consistent with those of previous studies. Nonetheless, the ABI5-regulated inhibition of seed germination and early seedling growth, to protect the plant against adverse conditions via regulation of development, has also been reported [42]. However, a previous study showed that seedlings germinated and developed normally in the absence of ABA in spite of ABI5 accumulation [43]. Therefore, it is reasonable to suggest that *PmABI5* may improve stress resistance by enhancing seedling growth and passing through the stress-sensitive period as early as possible.

Plant-specific NAM/ATAF/CUC (NAC) transcription factors (TFs) were initially associated with development [44] but are now being increasingly appreciated for the roles they play in stress responses and senescence [45,46]. In this study, *PmABI5* was found to regulate the promoter region of *PmNAC1*, which in turn results in the regulation of *PmNAC1* expression; additionally, the NAC transcription factor family was shown to be involved in salt stress response as well as improving crop stress resistance in many plants. The three subgroup III genes, namely, *ANA019*, *ANAC055*, and *ANAC072* (*RD26*), are induced by drought, high salinity, and the hormones abscisic acid (ABA) and jasmonic acid (JA) [47]. The overexpression of the three NAC TFs results in the upregulation of several stress-inducible genes and improved drought tolerance [48], and *ANAC019* and *ANAC055* function as positive regulators of JA signaling defense responses [49]. Collectively, the molecular, morphological, and physiological evidence clearly indicates that *PmABI5* confers higher tolerance under salt stress conditions by regulating effector genes for increasing salt tolerance or root development in crops. Our study also found that ABI5-NAC modules may regulate the expression of abiotic-responsive genes, but further investigation of the downstream regulatory genes is still needed.

## 4. Materials and Methods

### 4.1. Plant Materials and External Characteristics of Seeds

**Figure 11:**
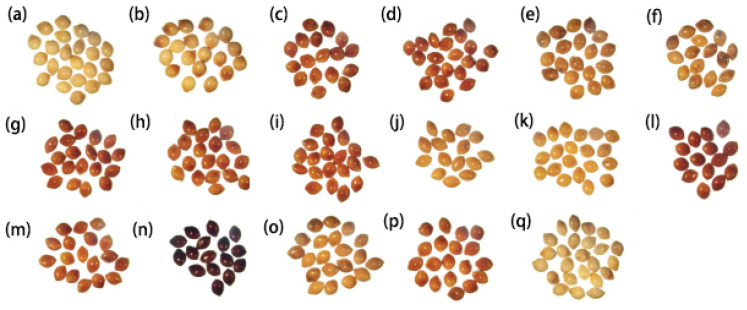
External seed morphological characteristics of the 17 broomcorn millet populations considered in this study: (**a**) Jinmi 4; (**b**) Jinmi 9; (**c**) Longmi 7; (**d**) Longmi 8; (**e**) Neimi 5; (**f**) Neimi 6; (**g**) Ningmi 10; (**h**) Ningmi 11; (**i**) Qingmi 1; (**j**) Qingmi 2; (**k**) Yumi 9 (Y9); (**l**) Yumi 8; (**m**) Tianmi 1; (**n**) Xingmi 1; (**o**) Yanmi 7; (**p**) Yanmi 8; (**q**) Yumi 1 (Y1).

To perform the seed germination experiments, we selected 17 broomcorn millet populations which represent commonly available cultivars in Northwest China (Table 5). An OLYMPUS microscope (Zoom Stereomicroscope SZ61/SZ51) was used to observe the seed color, size, shape, and skin (Figure 11).

On the basis of the preliminary experiments on germination rates, two varieties (Yumi 1 and Yumi 9) were selected for sequencing. Samples were originally collected from Yulin, Shaanxi, China. The population of Yumi 1 is a waxy broomcorn millet (seed coat color: white) with a glutinous taste due to the amylopectin content of starch in the endosperm. The other population was Yumi 9, which is a non-waxy broomcorn millet (seed coat color: yellow).

### 4.2. Salt Stress Treatment and Seed Germination Experiment

In total, 100 seeds were germinated on two pieces of filter paper in Petri dishes (9 cm × 9 cm × 1 cm). Seeds in germination dishes were pre-chilled (4 °C) for 2 d prior to germination, to aid in breaking seed dormancy as per the Association of Official Seed Analysts (AOSA) guidelines, and further cultured at 25 °C in continuous light. Germination conditions were set at 0 mM NaCl (only RO water) as control and 250 mM NaCl treatment.

Under suitable temperature conditions, the seeds first absorb water. Subsequently, the protoplasm inside the seed changes from a gel to a sol state, and enzymatic activity rapidly increases. The nutrients inside the seeds begin to transform and decompose from the endosperm or cotyledons and are transported to the radicle. In addition, germ cells were reconstructed, referred to as a sprout. After radicle elongation, white buds were revealed, which are called dew white. Seeds that reach this period are defined as germinated. The number of seeds that were germinated was counted and recorded daily when the seeds began to grow (i.e., from the beginning of the period of incubation 25 °C in continuous light).

### 4.3. RNA-Seq Sampling and Total Ribonucleic Acid (RNA) Preparation

The samples of Yumi 1 and Yumi 9, both cultured in 0 and 250 mM NaCl, were collected 0 and 3 h upon the emergence of dew white seeds following the onset of continuous light (each sample had three independent biological replicates). Samples were then rapidly frozen with liquid nitrogen and stored at −80 °C. There were 36 samples in total used for RNA-Seq and differential expression analyses.

Total RNA was extracted from seeds using the RNA prep pure polysaccharide polyphenol plant total RNA extraction kit (DP441) (TIANGEN, Beijing, China). RNA degradation and contamination were monitored on 1% agarose gels. RNA purity was checked using the NanoPhotometer^®^ spectrophotometer (IMPLEN, Westlake Village, CA, USA). RNA concentration was measured using Qubit^®^ RNA Assay Kit in Qubit^®^ 2.0 Fluorometer (Life Technologies, CA, USA). RNA integrity was assessed using the RNA Nano 6000 Assay Kit of the Agilent Bioanalyzer 2100 system (Agilent Technologies, Santa Clara, CA, USA).

### 4.4. Complementary DNA (cDNA) Library Preparation for Transcriptome Sequencing

A total amount of 1.5 µg RNA per sample was used as input material for the RNA sample preparations. Sequencing libraries were generated using NEBNext^®^ Ultra™ RNA Library Prep Kit for Illumina^®^ (NEB, Ipswich, MA, USA) following the manufacturer’s recommendations, and index codes were added to attribute sequences to each sample. Briefly, mRNA was purified from total RNA using poly-T oligo-attached magnetic beads. Fragmentation was carried out using divalent cations under elevated temperature in NEBNext First Strand Synthesis Reaction Buffer (5×). First-strand cDNA was synthesized using random hexamer primer and M-MuLV Reverse Transcriptase (RNase H-). Secondstrand cDNA synthesis was subsequently performed using DNA Polymerase I and RNase H. Remaining overhangs were converted into blunt ends via exonuclease/polymerase activities. After adenylation of 3′ ends of DNA fragments, NEB Next Adaptor with hairpin loop structure was ligated to prepare for hybridization. To select cDNA fragments of preferentially 250~300 bp in length, the library fragments were purified with an AMPure XP system (Beckman Coulter, Beverly, CA, USA). Then, 3 µL USER Enzyme (NEB, MA, USA) was used with size-selected, adaptor-ligated cDNA at 37 °C for 15 min followed by 5 min at 95 °C prior to PCR. PCR was then performed using Phusion High-Fidelity DNA polymerase, Universal PCR primers, and Index (X) Primer. Finally, PCR products were purified (AMPure XP system) and library quality was assessed on the Agilent Bioanalyzer 2100 system.

### 4.5. Raw Data Assembly and Gene Functional Annotation

The adaptor sequences and low-quality sequence reads were removed from the datasets. Raw sequences were transformed into clean reads after data processing. These clean reads were then mapped to the reference genome sequence [50]. Only reads with a perfect match or one mismatch were further analyzed and annotated on the basis of the reference genome. Tophat2 tools software was used to perform mapping to the reference genome.

Gene function was annotated on the basis of the following databases: nr (NCBI non-redundant protein sequences); nt (NCBI non-redundant nucleotide sequences); Pfam (Protein family); KOG/COG (Clusters of Orthologous Groups of proteins); Swiss-Prot (a manually annotated and reviewed protein sequence database); KO (KEGG Ortholog database); GO (Gene Ontology).

### 4.6. Differential Expression Genes (DEGs) Analysis

Differential expression analysis of two conditions/groups was performed using the DESeq R package (1.10.1). DESeq provides statistical routines for determining differential expression in digital gene expression data using a model based on the negative binomial distribution. The resulting *p*-values were adjusted using Benjamini and Hochberg’s approach for controlling the false discovery rate. Genes with an adjusted *p*-value < 0.05 found by DESeq were denoted as differentially expressed.

Gene Ontology (GO) enrichment analysis of the differentially expressed genes (DEGs) was implemented using the GOseq R package-based Wallenius noncentral hypergeometric distribution [51], which can adjust for gene length bias in DEGs.

KEGG [52] is a database resource for understanding high-level functions and utilities of the biological system, such as the cell, the organism, and the ecosystem, from molecular-level information, especially large-scale molecular datasets generated by genome sequencing and other high-throughput experimental technologies (http://www.genome.jp/kegg/, accessed on 15 November 2018). We used KOBAS [53] software to test the statistical enrichment of differential expression genes in KEGG pathways.

Heatmap used the R package pheatmap, which uses default parameters. The combination of distance and algorithm is Euclidean distances and complete linkage.

### 4.7. Plant Seedling Stage Conditions and Stress Treatments

Millet seeds after disinfection were sown on Petri plates and incubated at 24 °C (8 h dark/16 h light) in a growth chamber. Seedlings at the two-leaf stage were transferred into vegetative soil matrix culture for four weeks. Millet seedlings were then treated with either 20% PEG6000, 100 μM ABA, 200 mM NaCl, 4 °C cold incubation, or 42 °C heat shock. Leaves were collected at 0, 3, 6, 9, 12, and 24 h after treatment and stored at −80 °C.

The *Arabidopsis* materials used in this study were in the Columbia (Col-0) background. After germinating in ½ Murashige and Skoog (MS) medium, wild-type (WT) plants and transgenic lines were grown in a growth chamber at a temperature of 22 °C, relative humidity of 65%, and with a 16 h light/8 h dark photoperiod.

### 4.8. Quantitative Real-Time PCR

Total RNA (1 μg) from relative samples was used to generate complementary DNA (cDNA) templates using the PrimeScript™ RT reagent Kit with gDNA Eraser (Perfect Real Time) (Takara, RR047Q). Gene-specific primers and TB Green™ Premix Ex Taq™ II (Tli RNaseH Plus) (Takara, RR820Q) were used for QPCR on StepOne System (Applied Biosystems, Darmstadt, Germany) according to the manufacturer’s instructions. Gene-specific primers were designed by NCBI (https://www.ncbi.nlm.nih.gov/tools/primer-blast/index.cgi?LINK_LOC=BlastHome, accessed on 13, November 2019) (Table 6). PCRs included 2.0 μL of first-strand cDNA (1:10 diluted cDNA), 5 μL of SYBR Mix, and 10 nM of each primer in a final volume of 10 μL. The primers were designed in non-polymorphic regions across accessions. For each gene, we used cDNA samples of three biological replicates per accession as a template for QPCR.

Quantification of gene expression was carried out by the relative quantification method (2^−ΔΔCT^ method) implemented in StepOne software v2.3. Since the efficiencies of the several primers were not identical in the three-millet group, we corrected the relative quantification results according to the efficiency of each of the genes in each of the groups by StepOne software v2.3 on the basis of the following equations: Efficiency = 10 (−1/slope) for each primer and RQ = Etarget − (Ct treatment − Ct control)/Endogenous − (Ct treatment − Ct control).

### 4.9. Subcellular Localization and BiFC Assay

The full-length *PmbZIP33*, *PmbZIP131, PmbZIP125*, *PmABI5*, *PmbZIP97*, and *PmbZIP118* ORFs were cloned into the pCAMBIA2300-eGFP vector to generate the PmbZIPs:eGFP fusion constructs. PmbZIPs:eGFP were transformed into *Nicotiana benthamiana* leaves via *Agrobacterium*-mediated transformation.

In the BiFC assays, the full-length PmbZIPs (including PmABI5) were fused with the C-terminal of C-YFP via the pCAMBIA2300-VYCE vector and PmbZIPs (including PmABI5) were also fused to the N-terminal of N-YFP using the pCAMBIA2300-VYNE vector, recombinant plasmid pairwise combination, and via *Agrobacterium*-mediated co-transformation into *Nicotiana benthamiana* leaves. All the leaves were collected at 48 h after transformation, and the fluorescent signal was detected using confocal microscopy (IX83-FV1200, Olympus, Philadelphia, PA, USA).

### 4.10. Construction of Transgene Vector and Plant Transformation

The full-length PmbZIP1, PmbZIP2, PmbZIP3, PmbZIP4, PmbZIP5, and PmbZIP7 ORFs were cloned into the pCAMBIA2300-eGFP vector to generate the PmbZIPs:eGFP fusion constructs. The parental line of *Arabidopsis thaliana* L. was Col-0, the overexpressing lines Floral Dip were used for transformation. The parental line of *Oryza sativa* L. was Nipponbare, and the method used was as previously described [54].

Plant samples were imaged, and the surface area, average root diameter, length, and volume of roots were measured by EPSON V700 Root Analysis System (Seiko, EPSON Crop, Nagno, Japan), and analyzed by WinRhizo (Regent Instruments Inc., Quebec, QC, Canada).

### 4.11. Y1H Assays

Yeast one-hybrid assay, used to identify the promoter binding site, was performed with yeast strain Y1HGold cells (Clontech). Three copies of G-box (CACGTG) and three copies of its mutant sequence g-box (CATTTG) were cloned into the pAbAi vector (Figure 11a,b). pGbox-ABAi and pgbox-ABAi were transformed into the Y1HGold strain after linearization, Gbox-ABAi and gbox-ABAi reporter strains were obtained by homologous recombination. At the same time, fusion of full-length *PmABI5* to the C-terminal GAL4 activation domain using the pGADT7 vector. Fusion vector was transformed into two reporter strains and the control comprised the empty pGADT7 vector. All yeasts were tested on SD/−Ura/−Leu and SD/−Ura/−Leu medium with 800 ng/mL AbA.

## Figures and Tables

**Table 1:** Seed germination rate of various broomcorn millet varieties under salt treatment.

Variety	Treat	Seed Germination Rate							
		Day 1	Day 2	Day 3	Day 4	Day 5	Day 6	Day 7	Day 8
Jinmi 4	CK	0%	0%	59%	73%	76%	83%	85%	88%
	NaCl	0%	0%	2%	13%	16%	18%	24%	24%
Jinmi 9	CK	0%	0%	35%	50%	56%	57%	57%	57%
	NaCl	0%	0%	7%	24%	26%	26%	26%	26%
Longmi 7	CK	0%	0%	51%	65%	77%	81%	84%	85%
	NaCl	0%	0%	0%	13%	20%	23%	25%	26%
Longmi 8	CK	0%	0%	16%	38%	44%	54%	56%	58%
	NaCl	0%	0%	3%	16%	23%	24%	26%	26%
Neimi 5	CK	0%	0%	36%	43%	44%	51%	51%	52%
	NaCl	0%	0%	0%	8%	14%	15%	15%	15%
Neimi 6	CK	0%	0%	40%	53%	61%	63%	64%	64%
	NaCl	0%	0%	2%	14%	21%	24%	26%	26%
Ningmi 10	CK	0%	0%	20%	35%	39%	42%	44%	45%
	NaCl	0%	0%	1%	5%	17%	19%	23%	23%
Ningmi 11	CK	0%	0%	53%	62%	67%	71%	74%	75%
	NaCl	0%	0%	1%	1%	1%	1%	1%	1%
Qingmi 1	CK	0%	0%	9%	22%	23%	33%	35%	35%
	NaCl	0%	0%	1%	5%	6%	6%	6%	6%
Qingmi 2	CK	0%	0%	50%	51%	51%	51%	51%	51%
	NaCl	0%	0%	16%	23%	23%	23%	25%	25%
Yumi 9	CK	0%	0%	97%	100%	100%	100%	100%	100%
	NaCl	0%	0%	50%	78%	80%	80%	80%	80%
Yumi 8	CK	0%	0%	31%	39%	54%	63%	68%	68%
	NaCl	0%	0%	0%	16%	26%	28%	38%	42%
Tianmi 1	CK	0%	0%	74%	80%	82%	82%	82%	82%
	NaCl	0%	0%	24%	41%	47%	47%	47%	49%
Xingmi 1	CK	0%	0%	22%	63%	76%	78%	79%	79%
	NaCl	0%	0%	0%	0%	1%	1%	2%	2%
Yanmi 7	CK	0%	0%	52%	92%	96%	96%	96%	96%
	NaCl	0%	0%	0%	3%	23%	27%	30%	31%
Yanmi 8	CK	0%	0%	55%	84%	94%	95%	95%	95%
	NaCl	0%	0%	0%	2%	11%	20%	24%	29%
Yumi 1	CK	0%	0%	37%	64%	69%	71%	74%	75%
	NaCl	0%	0%	2%	10%	22%	22%	22%	23%

Note: CK is seed germination in RO water, NaCl is seed germination in 250 mmol/L. On Day 1 and Day 2, seeds were pre-chilled (4 °C).

**Table 2:** Information of the sequencing data.

Sample ID	Clean Reads	Clean Bases	Q30	GC Content	Mapped Reads	Uniq. Mapped Reads
Y9_CK1	29,724,228	8,894,995,347	88.79%	62.95%	80.66%	79.15%
Y9_NA1	30,282,360	9,064,513,521	89.53%	63.13%	82.53%	81.01%
Y9_CK2	30,648,074	9,178,726,745	88.54%	58.36%	82.39%	80.08%
Y9_NA2	28,758,908	8,613,482,668	89.95%	58.54%	84.41%	82.22%
Y9_CK3	29,993,545	8,981,924,205	89.85%	58.21%	84.57%	82.51%
Y9_NA3	30,141,521	9,020,749,193	89.17%	58.29%	82.69%	79.96%
Y1_CK1	28,755,425	8,607,858,315	88.49%	60.93%	72.93%	71.51%
Y1_NA1	31,833,050	9,532,800,895	88.97%	61.87%	79.63%	78.08%
Y1_CK2	27,836,385	8,333,972,016	89.90%	58.94%	79.83%	77.98%
Y1_NA2	29,149,177	8,726,183,941	89.70%	59.85%	79.87%	77.73%
Y1_CK3	26,299,506	7,872,286,537	88.83%	58.50%	77.64%	75.49%
Y1_NA3	30,135,668	9,022,400,108	88.91%	58.98%	80.64%	78.25%

Note: Y9, Y1 represent Yumi 9 and Yumi 1, respectively. CK represents control, NA represents salt stress (250 mM NaCl). 1, 2, 3 represent the three periods.

**Table 3:** Summary of gene annotation information.

Database	Annotated Number	Percentage (%)	300 ≤ Length < 1000	Length ≥ 1000
COG	18,435	30.56	5529	12,720
GO	44,154	73.19	16,756	26,132
KEGG	17,925	29.71	6798	10,722
KOG	28,998	48.06	9803	18,747
Pfam	47,074	78.03	17,310	28,871
Swiss-Prot	37,227	61.70	13,031	23,556
eggNOG	56,482	93.62	22,693	31,930
Nr	59,971	99.40	24,734	32,843
All Annotated	60,331	100.00	24,955	32,893

**Table 4:** Statistics of differentially expressed genes.

Groups	Differentially Expressed Genes	Upregulated Genes	Downregulated Genes
Y9_CK1 vs. Y9_NA1	88	34	54
Y9_CK2 vs. Y9_NA2	1898	613	1285
Y9_CK3 vs. Y9_NA3	4316	2860	1456
Y1_CK1 vs. Y1_NA1	77	58	19
Y1_CK2 vs. Y1_NA2	425	97	328
Y1_CK3 vs. Y1_NA3	3051	1447	1604
Y1_CK1 vs. Y9_CK1	1182	718	464
Y1_CK2 vs. Y9_CK2	2442	954	1488
Y1_CK3 vs. Y9_CK3	1968	568	1400
Y1_NA1 vs. Y9_NA1	910	548	362
Y1_NA2 vs. Y9_NA2	1406	628	778
Y1_NA3 vs. Y9_NA3	1338	477	861

Note: Y9, Y1 represent Yumi 9 and Yumi 1, respectively. CK represents control, NA represents salt stress (250 mM NaCl). 1, 2, 3 represent the three periods. Y9_CK1 vs. Y9_NA1 refers to the Y9 genes found to be differentially expressed in NA treatment compared with CK at the first sampling period. The remaining labels show similar comparisons but for different treatments and periods as indicated.

**Table 5:** Seed characteristics of the 17 broomcorn millet populations considered in this study.

Variety	Serial Number ^1^	Seed Coat Color	Waxy or Non-Waxy	Hardness
Jinmi 4	a	white	waxy	fragile
Jinmi 9	b	white	waxy	fragile
Longmi 7	c	red	unknown	hard
Longmi 8	d	red	unknown	hard
Neimi 5	e	yellow	non-waxy	hard
Neimi 6	f	yellow	non-waxy	hard
Ningmi 10	g	red	unknown	hard
Ningmi 11	h	red	unknown	hard
Qingmi 1	i	red	unknown	hard
Qingmi 2	j	yellow	non-waxy	hard
Yumi 9	k	yellow	non-waxy	hard
Yumi 8	l	red	unknown	hard
Tianmi 1	m	red	unknown	hard
Xingmi 1	n	black	unknown	hard
Yanmi 7	o	yellow	non-waxy	hard
Yanmi 8	p	yellow	non-waxy	hard
Yumi 1	q	white	waxy	fragile

^1^ The serial number is used to annotate Figure 11.

**Table 6:** Primers of qRT-PCR for validation of the selected DEGs.

Gene Name	Description	Primers
*PmbZIP33*	F	CCTTGGCAATGGACTGATGC
*PmbZIP33*	R	GATGACAGGTCGCTGTACCC
*PmbZIP131*	F	ACTGTGAGCCCGGTGGATAC
*PmbZIP131*	R	CTCCCTCAAATGGGTACGGC
*PmbZIP125*	F	CAGCGTCAAGACGGTGGAC
*PmbZIP125*	R	GGAACTCCTCGAGCGTCATC
*PmABI5*	F	TCAAGAACAGGGAGTCTGCG
*PmABI5*	R	CCGTCCGAATGGATTCCTCA
*PmbZIP97*	F	ATTCCGCCATGAAGTCGAGG
*PmbZIP97*	R	GACTCCTGCATGGCTATGGG
*PmbZIP89*	F	CAAAGGAACGAACACGCAGG
*PmbZIP89*	R	GGCAAAGAGGTCGATGTCCA
*PmbZIP118*	F	GCAGCGGCGGATGATAAAAA
*PmbZIP118*	R	CAACTTGCCGGCTCATTCTC
*Actin*	F	GCGAGCTTCCCTGTAGGTAG
*Actin*	R	CGAACCCAGCCTTCACCATAC

## Data Availability

The data presented in this study are available on request from the corresponding author.

## Supplementary Materials

The following supporting information can be downloaded at: https://www.mdpi.com/article/10.3390/ijms23126448/s1.
