# Supplementary material for: The Identification of Broomcorn Millet bZIP Transcription Factors, Which Regulate Growth and Development to Enhance Stress Tolerance and Seed Germination
Source: Int J Mol Sci. 2022 Jun 9;23(12):6448. doi: 10.3390/ijms23126448 (PMC9224411; doi:10.3390/ijms23126448)
Supplement: Supplementary file 1 [file ijms-23-06448-s001.zip › ijms-1760977-supplementary.pdf]

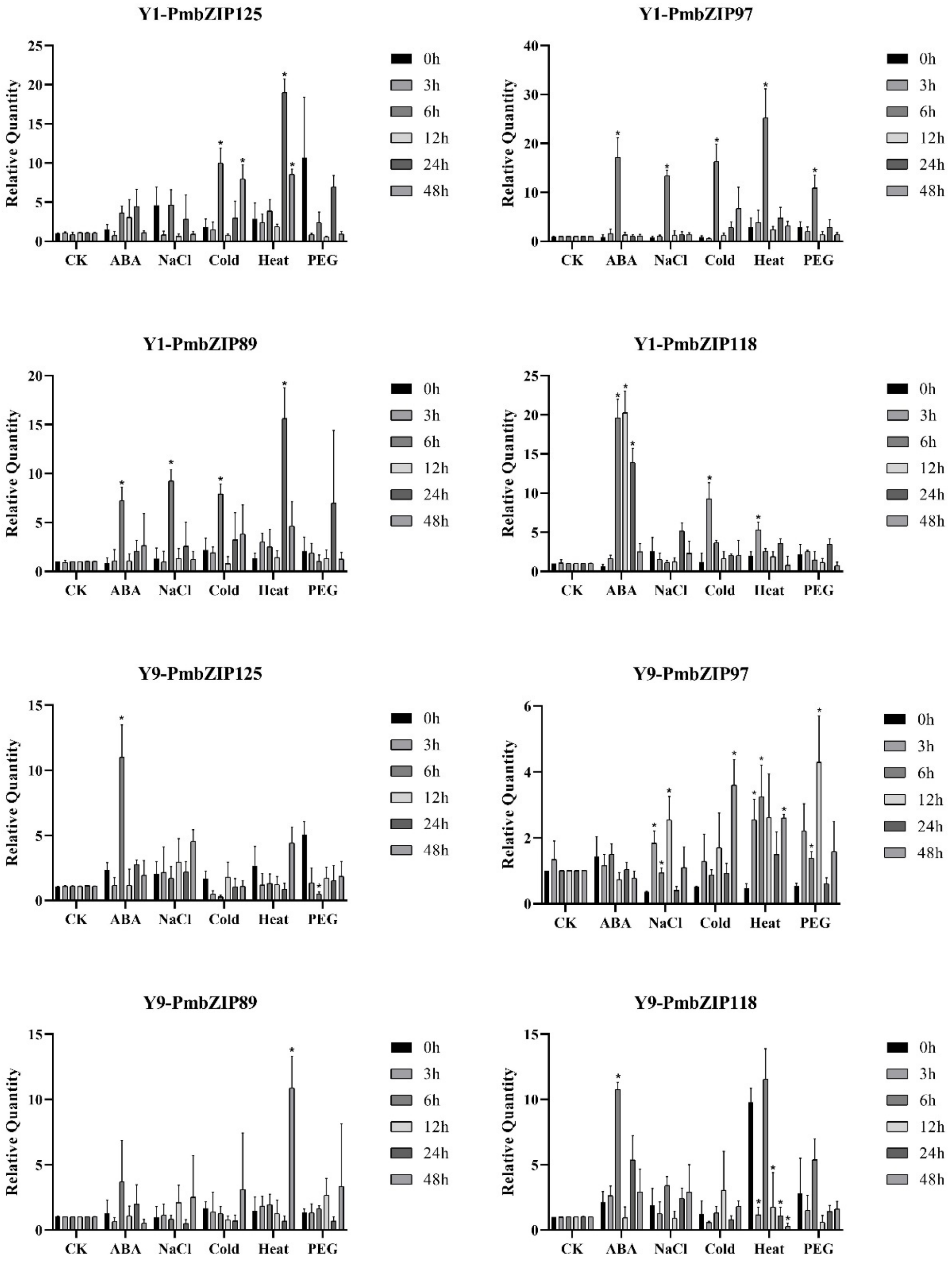

Figure S1. Expression pattern of *PmbZIP125,PmbZIP97,PmbZIP89* and *PmbZIP118*. Error bars are SD based on three biological replicates and three technical repeats. *: p value<0.05.

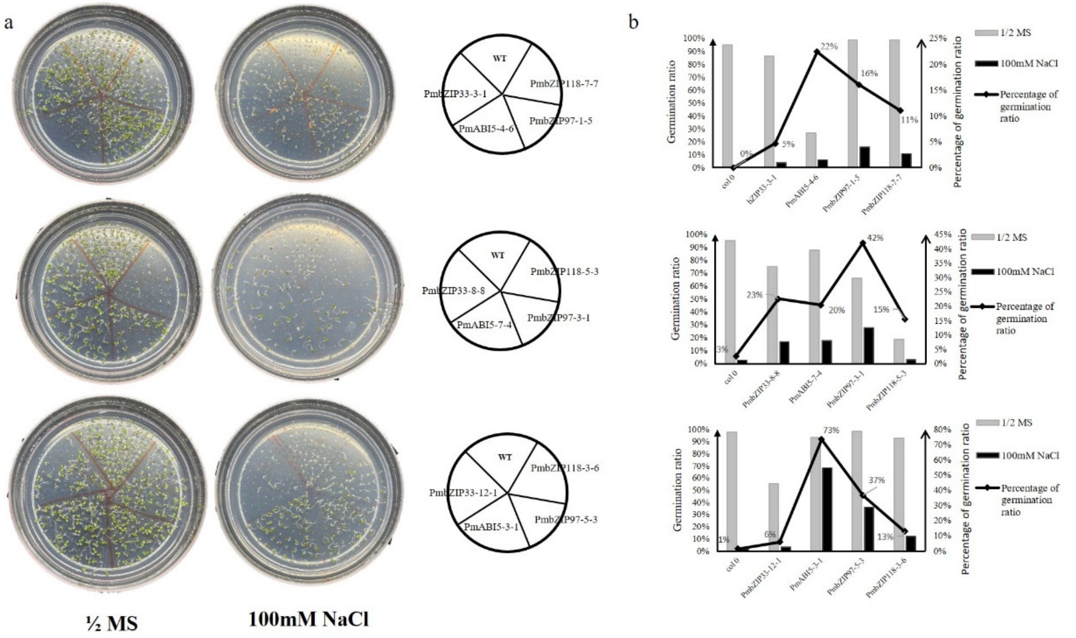

Figure S2. Germination of overexpression tansgene lines. (a) OE:*PmABI5* germination phenotype on Day 8. (b) OE:PmABI5 seed germination under different treatment. The left axis represents the germination ratio of different genotyes under ½ MS and 100mM NaCl treatment (colums). The right axis represents the percentage germination ratio under 100mM NaCl treatment compared to that under ½ MS condition. At least 50 seeds for each genotype were used for the germination assay.

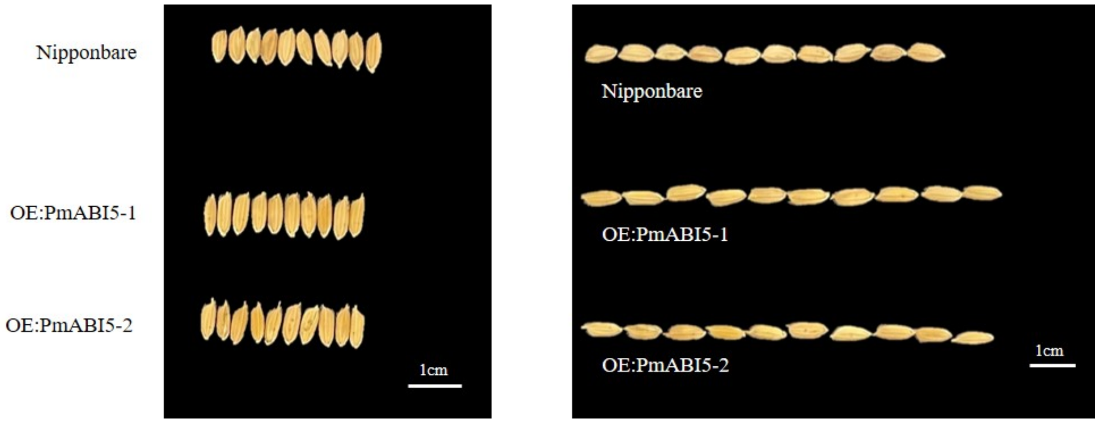

Figure S3. The seed size of PmABI5 rice overexpression tansgene lines
